# Assessment of Nasal-Brain-Targeting Efficiency of New Developed Mucoadhesive Emulsomes Encapsulating an Anti-Migraine Drug for Effective Treatment of One of the Major Psychiatric Disorders Symptoms

**DOI:** 10.3390/pharmaceutics14020410

**Published:** 2022-02-14

**Authors:** Hadel A. Abo El-Enin, Rasha E. Mostafa, Marwa F. Ahmed, Ibrahim A. Naguib, Mohamed A. Abdelgawad, Mohammed M. Ghoneim, Ebtsam M. Abdou

**Affiliations:** 1 Department of Pharmaceutics, College of Pharmacy, Taif University, Taif 21944, Saudi Arabia; hadel.a@tu.edu.sa; 2 Pharmacology Department, Medical Research and Clinical Studies Institute, National Research Centre, Giza 12622, Egypt; re.mostafa@nrc.sci.eg; 3 Department of Pharmaceutical Chemistry, College of Pharmacy, Taif University, Taif 21944, Saudi Arabia; i.abdelaal@tu.edu.sa; 4 Department of Pharmaceutical Chemistry, College of Pharmacy, Jouf University, Sakaka 72341, Saudi Arabia; 5 Department of Pharmacy Practice, Faculty of Pharmacy, AlMaarefa University, Ad Diriyah 13713, Saudi Arabia; mghoneim@mcst.edu.sa; 6 Department of Pharmaceutics, National Organization of Drug Control and Research (NODCAR), Giza 12622, Egypt; ebtsamabdou83@gmail.com

**Keywords:** mucoadhesive emulsomes, anti-migraine, psychiatric disorders symptoms, brain targeting

## Abstract

Migraine is one of the major symptoms of many psychiatric and mental disorders like depression and anxiety. Eletriptan Hydrobromide (EH) is a well-tolerated drug in migraine treatment, but suffers from low oral bioavailability and low brain targeting after oral delivery. New nasal mucoadhesive EH-emulsomes development could be a new means to direct the drug from the nose-to-brain to achieve rapid onset of action and high drug concentration in the brain for acute migraine treatment. Eletriptan mucoadhesive emulsomes formulations were prepared using thin-film hydration method and 2^3^ full factorial design was adopted to study different formulation factors’ effect on the emulsomes characters. The emulsomes were characterized for entrapment efficiency (EE%), zeta potential (ZP), particle size (PS), morphology, and ex-vivo permeation through the nasal mucosa. The selected formula was evaluated in mice for its in-vivo bio-distribution in comparison with EH intranasal and intravenous solutions. Drug targeting efficacy (DTE%) and nose-to-brain direct transport percentage (DTP%) were calculated. The optimization formulation showed a nanoparticle size of 177.01 nm, EE 79.44%, and ZP = 32.12 ± 3.28 mV. In addition, in-vitro permeability studies revealed enhanced drug permeability with suitable mean residence time up to 120 ± 13 min. EH-emulsomes were stable under different storage conditions for three months. In vivo examination and pharmacokinetic drug targeting parameters revealed EH transport to the CNS after EH nanoparticle nasal administration. Histopathology study showed no ciliotoxic effect on the nasal mucosa. From the results, it can be confirmed that the emulsomes formulation of EH proved safe direct nose-to-brain transport of EH after nasal administration of EH emulsomes.

## 1. Introduction

Migraine is a neurologic episodic headache attack, which directly affects over one billion people across all world regions [1]. It was the major symptom of many psychiatric and mental disorders such as depression and anxiety [2,3,4]. The main goal of migraine treatment is reducing the severity and duration with minimal side effects and developing a suitable formulation that can give quick onset of action [5,6]. Migraine headache is due to neurogenic inflammation of the trigeminal nerve in the cranial dura mater [7]. These central stimulations can participate in trigeminal neurons sensitization and activation [8].

Eletriptan Hydrobromide (EH), a second generation triptan, is a well-tolerated drug in migraine treatment [9]. It acts through the reduction of blood vessel swelling, associated with the head pain of a migraine attack, surrounding the brain. Although there is good absorption of EH following oral route administration, it is liable to first-pass metabolism resulting in low mean absolute oral bioavailability of about 50% [10]. Furthermore, its absorption is inhibited by a p-glycoprotein (P-gp) substrate, which reduces its blood–brain barrier (BBB) permeability by about 40-fold, necessitating high oral-dose delivery [8].

The intranasal route introduces the drugs directly to the brain by direct neuronal transport, via olfactory and trigeminal nerves. Nasal route is considered a non-invasive brain targeting route of administration. It can overcome the oral route limitations: first-pass metabolism effect and limited -BBB passing [11,12]. In addition, the intranasal route is known to impart rapid onset of action and high brain drug concentration, the matter which is needed for acute migraine treatment [13]. Despite the intranasal route advantages, the rapid clearance of the applied preparation by mucociliary clearance and the shorter residence time and enzymatic degradation are the main associated limitations. Mucoadhesive nano-vesicular carriers help in resolving these limitations as they helped in prolonging the nasal mucosa retention and improve cellular uptake and intracellular drugs disposition. Additionally, they could increase drug availability at different brain sectors [14] as well as, offer a higher drug loading and protection against enzymatic activity [15,16,17].

Emulsomes are defined as colloidal carriers containing solid or semisolid inner core, stabilized by phospholipids in high concentration, in the form of o/w emulsions [18]. They can combine the advantages of both emulsions and liposomes in addition to some close features to lipospheres and solid-lipid nanoparticles [19]. An important character of emulsomes is their internal core, which is formulated of a lipid in a solid-state, rather than an oil-in-fluid phase, that remains solid at 25 °C and has solid to liquid phase transition temperature near to physiological temperature [20]. They can be loaded with the water-soluble drugs in the aqueous compartments of the external phospholipid layers, while hydrophobic drugs can be loaded within the inner lipid core [19]. This bilayer structure improved the system’s stability. Additionally, emulsomes with their characteristic small size can provide site-specificity and accordingly, increase drug concentrations at targeted tissues [21]. Emulsomes may be used for different routes of drug delivery such as parenteral, ocular, oral, rectal, intranasal, vaginal, or topical delivery. Coating of emulsomes with a mucoadhesive polymer, trimethyl chitosan, is expected to increase their attachment force and time to the nasal mucosa and thus enhance drug permeation [18].

Hence, the goal of this project is to develop stable EH encapsulated emulsomes coated with mucoadhesive polymer aiming at a direct nose-to-brain targeting strategy to enhance migraine treatment. Suitable formulation components were selected after extensive investigations, then the developed formulation was characterized and optimized. The assessment of the in-vitro permeation from the prepared mucoadhesive emulsomes through nasal mucosa was performed in addition to the assessment of the brain-targeting efficiency of the prepared emulsomes through in-vivo study in rats. Evaluation of the histological effect of applying EH emulsomes formulation on the nasal mucosa was done to establish the safety and efficacy of EH in the treatment of migraine to ensure effective treatment of the major symptom of many psychiatric and mental disorders.

## 2. Materials and Methods

### 2.1. Materials

EH was obtained as a gift from Pfizer, Cairo, Egypt. Phosphatidylcholine (PC) from soybean lecithin, containing not less than 94% (PC) was obtained from Alfa Aesar (Kandel, Germany). Compritol^®^ 888 ATO (CA) was obtained as a gift from GATTEFOSSE, Saint-Priest, France. Glyceryl mono-stearate (GMS), Tripalmitin (TP), Trilaurine (TL), Stearic acid (SA), Triton X100, and Tween 80 were purchased from El-Gomheria Co., Cairo, Egypt. Trimethyl chitosan (TMC) of low molecular weight, was purchased from Sigma Aldrich, Cairo, Egypt. All other chemicals were of analytical grade and were purchased from El-Gomhoria Co., Cairo, Egypt.

### 2.2. Methodology

#### 2.2.1. Solubility Study of EH in Different Solid Lipids

Eletriptan Hydrobromide (EH) solubility in different solid lipids namely: Compritol^®^ 888 ATO (CA), Glyceryl mono-stearate (GMS), Tripalmitin (TP), Trilaurine (TL), and Stearic acid (SA) was assessed as follows.

An excess amount of EH was added to 3 g of each lipid into a screw-capped vial and mixed using vortex (BV1000 BenchMixer™, Sayreville, NJ, USA). The mixture was shaken for 2 days using a thermostatically controlled shaker (PSU-20i Orbital Multi-Platform Shaker, Thomas Scientific, Swedesboro, NJ, USA) at 70 ± 2 °C.

The mixture was centrifuged at 3000 rpm for 20 min using a centrifuge with an integrated heating system adjusted at 70 ± 2 °C (Remi Laboratory Centrifuge R32A, Remi Equipment, Bombay, India) then filtered using a 0.45 filter membrane (PVDF, Millipore, County Cork, Ireland). The whole filtration assembly was kept at 70 °C in a controlled oven for a period of 20–30 min to prevent lipid solidification at room temperature. From the filtered supernatant, an accurately weighed amount was taken and dissolved in 10 mL methanol and measured after suitable dilution (s) using a previously developed and validated HPLC method [22]. In brief, HPLC (Agilent 1100) with Thermo-C18 analytical column (5 µm; 150 × 4.6 mm) was used at 35 °C. The mobile phase was composed of methanol and water in the ratio of 35:65. The sample volume of 20 µL was injected and run at a flow rate of 1 mL/min with detection wavelength of 227 nm. EH concentration in different solid lipids was determined using a formerly established calibration curve of EH in methanol.

#### 2.2.2. Experimental Design

Design Expert 10.0.1 Stat Ease.Inc. software (Minneapolis, MN, USA) was used to design the experimental runs using full factorial 2^3^ design. Three independent factors were screened, including phosphatidylcholine: compritol (PC: CA) molar ratio. (X1), EH: total lipids molar ratio (X2), and trimethyl chitosan concentration (TMC) *w*/*v*%. Each was screened at two levels. The high-level factor was coded as +1 while the low level was coded as −1, to give a design with minimum 8 formulations in addition to triple center points (mid-level) of independent factors for improved statistical significance (Table 1). The response variables were entrapment efficiency (EE%), particle size (PS), zeta potential (ZP), permeability coefficient (Kp), and residence time (RT). Based on the predicted and adjusted R^2^, the best fitting model for each response parameter was selected using statistical analysis. Analysis of variance (ANOVA) was implemented for statistical analysis of the responses, and statistical significance was at *p* = 0.05.

#### 2.2.3. Preparation of EH-Loaded Mucoadhesive Emulsomes

EH-loaded mucoadhesive emulsomes were formulated by using the thin-film hydration method formerly described by Zhou and Chen, 2015 [23] with slight modification. EH, CA, PC, and Cholesterol were dissolved in a mixture of chloroform: ethanol (2:1) in a round–bottom flask. The flask was rotated in a water bath at 150 rpm under reduced pressure where the temperature was kept at 70 ± 2 °C until the organic solvent was completely evaporated and a thin film was formed. A total of 10 mL hydration medium (consisting of TMC and Tween 80 (1% *v*/*v*) in water, previously heated to 70 ± 2 °C, was used as a hydration medium with the aid of small glass beads (8 small glass beads, each with a diameter of 4 mm) to facilitate the film hydration until suspension is formed. After cooling to room temperature, the mixture was sonicated using a probe sonicator (SonifierVR 250 Branson, MO, USA) in an ice-bath for three cycles of 5 min (output 2, 40-watt, constant duty cycle) with 5 min intervals between cycles. The emulsomal mixture was stored at 4 °C for further evaluation. The detailed composition of EH-loaded emulsomes is shown in Table 2. One mole of: PC = 314 g, CA = 1060 g, EH = 463 g. T. lipids amount was kept constant at one g for each formulation. Cholesterol was incorporated in all formulations at a constant amount of 100 mg, while Tween 80 was used in concentration (1% *v*/*v*) of the hydration medium.

#### 2.2.4. Evaluation of EH-Loaded Mucoadhesive Emulsomes

Entrapment Efficiency (EE%) and Drug Loading Capacity (DLC) determination of EH-loaded mucoadhesive emulsomes were evaluated for their EE% ratio through the direct method. EH-loaded mucoadhesive emulsomes were separated from the un-entrapped drug through a cooling centrifuge at 10,000 rpm for 45 min. A determined amount of the prepared emulsomes was disrupted completely into 10 mL 1% Triton X100 and 20 µL injected into a HPLC system for determination of EH concentration using a previously developed and evaluated method [22]. EE% was calculated using the following equation:

EE% = EH entrapped amount/EH initial amount × 100
DLC was determined by the following Equation [24]:
DCL = (Total entrapped EH-free EH) weight of emulsomes
The experiment was done in triplicates and results were expressed as mean ± SD

##### Particle Size Analysis

The particle size of the prepared EH-loaded mucoadhesive emulsomes was determined by dynamic light scattering (Zeta-sizer Nano ZS-90, Malvern Instruments, Worcestershire, UK) after suitable dilution using distilled deionized water. All measurements and analytical settings were controlled using standard operating procedures. All measurements were carried out in triplicate, and the mean and standard deviation were calculated.

##### Zeta Potential Determination

Zeta potential of the prepared EH-loaded mucoadhesive emulsomes was measured under regular operating circumstances using a Zeta-sizer (Zeta-sizer Nano ZS-90, Malvern Instruments, Worcestershire, UK). Distilled deionized water was used to dilute the samples. The results are described as mean values (*n* = 3) ± SD.

#### 2.2.5. Permeation Study of EH from the Prepared Mucoadhesive Emulsomes through the Nasal Mucosa

For this permeation study, sheep nasal mucosa, provided by the local slaughterhouse, was used as a membrane. The study was conducted using Franz diffusion apparatus (Franz diffusion cell, Hanson Research Corporation (HRC), Variel Avenue, Chatsworth, CA, USA) using phosphate buffered saline (pH 7.4) medium [25].

##### Nasal Mucosa Preparation

Less than 1 h after slaughtering the sheep, the nasal mucosa, except the septum part, was isolated and the fatty tissues and different tissues were removed gently. Obtained mucosa was washed gently with distilled water and kept into isotonic saline at −20 °C till use [26]. The experiment was done in compliance with the expectations for animal care and use/ethics committees set out by the Association for Assessment and Accreditation of Laboratory Animal Care International.

##### Apparatus Assembly

The prepared mucosal specimen of appropriate size having an effective surface area of 1.55 cm^2^ and 0.12 cm thickness was used for permeation study [27]. The specimen was mounted on the diffusion cell between the donor and receptor compartment, which was charged with phosphate buffered saline (pH 7.4) for 30 min at room temperature. The mucosal surface was kept facing the donor chamber and the dorsal side was kept facing the receptor chamber. The receptor compartment was filled with appropriate volume (7.5 mL) of phosphate-buffered saline (pH 7.4). The whole assembly was incubated in a thermostatically controlled water bath with temperature adjusted at 37 ± 0.5 °C for approximately 10 min to stabilize prior to loading of the test sample. The receptor chamber’s solution was continually stirred using Teflon-coated magnetic bar at a constant rate of 25 rpm [28].

Aliquots of 1 mL of the receptor medium were removed at appropriate time intervals and immediately replaced with fresh medium pre-heated to 37 ± 0.5 °C. Samples were injected into an HPLC system for the determination of EH concentration. Each experiment was performed in triplicates and obtained results were expressed as mean ± SD.

The steady-state flux (Jss, mg/cm^2^/h) was computed from the quantity of EH, which permeated through the nasal mucosa (Q) divided by the membrane surface and the time duration. The permeability coefficient (Kp, cm/h) was calculated from Jss and the initial drug concentration (C_0_) as given below [29].

Kp = Jss/C_0_

##### Determination of the Residence Time (RT) of the Prepared EH-Loaded Mucoadhesive Emulsomes

The residence time of the prepared mucoadhesive emulsomes was assessed by a formerly mentioned method [30]. Agar plates, 1% *w*/*w* agar dissolved in phosphate buffer saline (PBS) 6.4, were prepared and 100 mg of each formulation were centered on the agar plate, and to assure the sample, attachment was left for 5 min. After that, the agar plates were attached to USP disintegration test apparatus (2901, ElectronicsIndia Co., Haryana, India) and moved up and down in PBS pH 6.4 at 37 ± 0.5 °C. The residence time in seconds (RT) was recorded at the time the formulations were completely detached from the agar. The experiment was done in triplicates for each formulation and obtained results were expressed as mean ± SD.

#### 2.2.6. Optimization of EH-Loaded Mucoadhesive Emulsomes

After the all responses were analyzed, constraints (goals) on dependent (responses) and independent variables (factors) were applied using Design-Expert^®^ software (Minneapolis, MN, USA) to obtain the optimized formulation.

#### 2.2.7. Morphological Evaluation

The morphological evaluation of the optimized EH-loaded emulsomal formulation was conducted by (TEM) (JEOL JEM1230, Tokyo, Japan) operating at an accelerating voltage of 80 kV. A drop of the sample was deposited on a carbon-coated copper grid to form a thin film. Before the film dried on the grid, it was negatively stained with 1% phosphotungstic acid (PTA). A drop of the staining solution was applied to the film, and the excess was drained off with filter paper. The grid was allowed to air dry thoroughly and samples were viewed in a transmission electron microscope.

#### 2.2.8. Stability Study of the Optimized Formulation

Samples of the optimized formulation were stored in a tightly closed vial at two different conditions; the first was at 4 °C and controlled humidity of 75% RH and the second at 25 ± 2 °C and controlled humidity of 75% RH. After 3 months, samples were taken and stability study was assessed through EE%, particle size, and Zeta potential measurements in addition to visual examination of the formulation for any color change or sedimentation [31].

#### 2.2.9. In-Vivo Biodistribution Studies

Formulation (F10) was selected, based on statistical optimization, to be evaluated in-vivo for its biodistribution in mice in comparison with EH nasal and intravenous solutions.

##### Animals

Adult male Swiss Albino mice (*Mus musculus*) weighing 25–30 g provided by the National Research Centre’s animal house colony (NRC, Egypt) were used in the current study. Animals were offered free access to a standard diet and tap water ad libitum. They were housed for one week before the experiment for acclimatization at room temperature and natural light/dark conditions. The National Institutes of Health Guide for the Care and Use of Laboratory Animals (NIH Publications No. 8023, revised 1978) and the National Research Center–Medical Research Ethics Committee (NRC-MREC) for the use of animals were followed in this investigation. The study protocol was approved by the ethical committee of Al-Taif University, KSA, Approval number: 43-021, 29-9-2021.

##### Experimental Protocol

Ninety-six mice were randomly allocated into three equal groups (32 mice each) and were administered to the tested treatments as follows:

- Group 1: Intranasal (i.n) aqueous solution of EH (equivalent to 1 mg/kg body weight) (10 µL in each nostril).
- Group 2: Intravenous (i.v) aqueous EHsolution (equivalent to 1 mg/kg body weight) that was injected through the lateral tail vein of the mice.
- Group 3: The selected EHformulation at a dose equivalent to 1 mg/kg body weight was instilled into the nose of the mice fixed in a prostrate position.

Intranasal administration (i.n) was done using a micropipette at a constant volume 10 µL in each nostril (Robfield-Gmbtt Kobenicker, Strabe 320, Düsseldorf, DeutschLand) attached to 0.1 mm polyethylene tube and was performed on rats laid on their backs in a slanted position gently with allowing the animals to inhale all the preparation [32].

##### Biochemical Analysis

The experiments extended for 8 h, and at each predetermined time interval (10, 20, and 30 min, 1, 2, 4, 6, and 8 h), blood samples were obtained under anesthesia from each group (four mice) via retro-orbital venous plexus into-heparinized test tubes. Blood samples were collected and left to remain at room temperature for 10 min before being centrifuged at 4 °C using a cooling centrifuge (Laborezentrifugen, 2k15; Sigma, Darmstadt, Germany) at 3000 r/min for 10 min and plasma was obtained. Mice were then sacrificed by cervical dislocation under anesthesia. Brains were dissected out, washed three times with normal saline, and then freed from any adhering tissues or fluids and weighed. Brains were homogenized separately with normal saline using a tissue homogenizer (Thomas Scintifica, Swedesboro, NJ, USA). Plasma samples and brain homogenates were stored at 20 °C until use.

EH concentration in both plasma and brain samples was measured using a standardized and validated high-performance liquid chromatography method [33]. EH brain targeting after intranasal administration was estimated by calculating different mathematical parameters [34] as drug targeting index (DTI), which is the ratio of the value of the area under the curve in the brain to the area under the curve in the blood AUC_brain_/AUC_blood_ after intranasal administration to that after intravenous administration. The drug targeting efficacy (DTE%) and nose-to-brain direct transport percentage (DTP%) were calculated as follows [32]:

(1) $$\mathrm{DTE}\%=\left(\frac{\frac{\mathrm{AUC}\;\mathrm{brain}}{\mathrm{AUC}\;\mathrm{blood}}\;i.n}{\frac{\mathrm{AUC}\;\mathrm{brain}}{\mathrm{AUC}\;\mathrm{blood}}\;i.v}\right)\times 100$$

(2) $$\mathrm{DTP}\%=\left(\frac{B\;i.n-\mathrm{Bx}}{B\;i.n}\right)\times 100$$

where Bx = (Bi.v/Pi.v) × Pi.n, Bx is the brain AUC _(0–8)_ fraction contributed by systemic circulation through the BBB following intranasal administration.

Bi.v and Bi.n are the AUC _(0–8)_ (brain) following intravenous administration and intranasal administration, respectively, while Pi.v and Pi.n are the AUC _(0–8)_(blood) following intravenous and intranasal administration, respectively.

#### 2.2.10. Histopathological Examination

To study the effect of the selected EH emulsomes formulation on the integrity of the nasal mucosa in mice, the mice noses were separated at the end of the in vivo biodistribution study and specimens from the nasal mucosa were collected. Nasal specimens were fixed in 10% neutral buffered formalin before being washed, dehydrated, clarified, and embedded in paraffin. For histological investigation, the paraffin blocks were sectioned at 4–5-micron thickness and stained with hematoxylin and eosin. The sections were examined under ×400 magnification using a binocular Olympus CX31 microscope (Motic1 BA210, Hicksville, NY, USA).

#### 2.2.11. Statistical Analysis

The data from all experiments were reported as the mean value ± SD. One-way analysis of variance (ANOVA) was used to assess the statistical data, and *p* < 0.05 was judged significant with 95% percent confidence intervals.

## 3. Results and Discussion

### 3.1. Solubility Test

**Figure 1:**
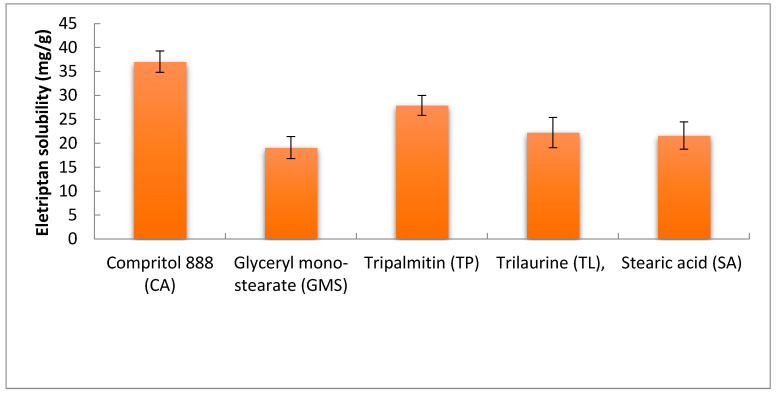
Solubility of EH in different solid lipids.

EH was found to have the highest solubility in Compritol (CA), 37.04 ± 2.23 mg/g, followed by Tripalmitin (TP), 27.90 ± 2.10 mg/g, Figure 1. The high solubility capacity of CA may be related to its composition as it is a chemical mixture of diacylglycerols, mostly dibehenylglycerol, and varying amounts of monoglycerols and triacylglycerols synthesized by the esterification of glycerol with behenic acid. For a drug to be formulated into lipid-based vesicles, it is important to be sufficiently solubilized in the lipid phase for successful encapsulation. Therefore, CA was selected as a liquid lipid for the formulation of EH emulsomes along with phosphatidylcholine as a solid lipid characterized by its biocompatibility and lower drug leakage tendencies [35].

### 3.2. EH-Emulsomes Formulation

The main goal of emulsomes formulation is to obtain formulae with uniform nanoparticle size, maximum stability, and entrapment efficiency. All the selected lipids should be biocompatible, biodegradable, non-toxic, and with appropriate concentrations. The prepared emulsomes should not be irritant or cause nasal mucosa damages [36].

Cholesterol was incorporated in all formulations to act as a stabilizing agent as it can induce the liquid crystal phase formation by altering the core packaging structure. Also, it can stabilize the outer phospholipid layers resulting in entrapment efficiency increasing and drug leakage reduction [23]. Tween 80 also was used in 1% *v*/*v* to improve the bilayer formation of the emulsomes and improve the carrying capacity of the lipid particles, even in the presence of an aqueous solution of the drug [37].

In this study, trimethyl chitosan (TMC), which is the simplest form of quaternized chitosan, was used as it is water-soluble over a wide pH range, has higher bio-adhesive and permeation properties than chitosan [38]. Results of DLC % of the prepared formulations are recorded in Table 2.

### 3.3. Evaluation of the Prepared Emulsomes

#### 3.3.1. Effect of Different Factors on Dependent Variables

Table 2 shows the different prepared formulae composition with different variables (PC: CA molar ratio, EH: T. lipids molar ratio and TMC concentration) and their effect on the PS, EE, ZP, KP, and RT results of eleven EH-emulsomes formulations. All factors have a significant effect on the tested responses, with a non-significant lack of fit, and follow a linear model with an R^2^ value > 0.97. Statistical analysis results are shown in Table 3 and the final equations in terms of coded factors are represented in Table 4.

**Figure 2:**
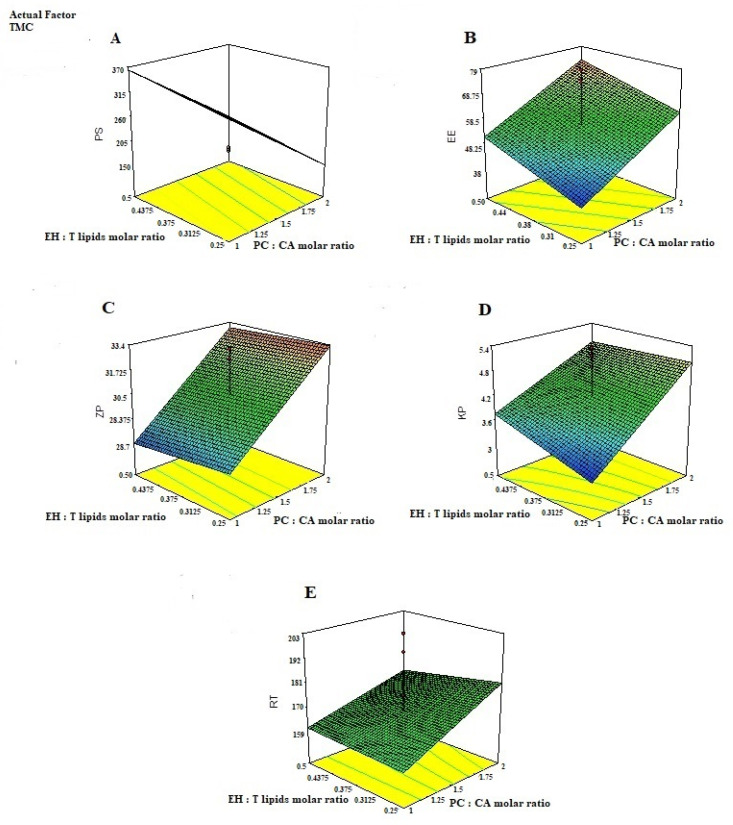
3D-plots response for studying the effect of PC: CA molar ratio, EH: T. lipids molar ratio, and TMC conc on; (**A**) the particle size PS, (**B**) entrapment efficiency, (**C**) Zeta potential (ZP), (**D**) Permeability Coefficient (Kp), and (**E**) Residence time (RT) of EH-mucoadhesive emulsomes.

ANOVA results indicated that all studied linear terms (PC: CA molar ratio, EH: T. lipids molar ratio, and TMC Conc), corresponding to the investigated variables, have a significant effect on all the tested responses at a 95% level of significance. The interaction between TMC concentration and either PC: CA molar ratio or EH: T. lipids molar ratio was also found to be significant at the same level. Figure 2 illustrates the response surface plots for the effects of the investigated variables on various responses.

The average EE% of the prepared EH emulsomes ranged from 79.73 ± 2.96 to 35.42 ± 3.84%, Table 2. According to the expressed equation (Table 4), it was found that all factors have a significant effect on the EE% of the prepared emulsomes. Higher PC: CA molar ratio resulted in higher EE% at the same EH: T. lipid molar ratio and TMC concentration. Higher PC amount could help in the formation of multilayers around the lipid core, permitting EH intercalation into these bilayers [18,19] in addition to its interlocation into the solid lipid core. Higher EH amounts resulted in higher EE% at constant PC: CA ratio and TMC concentration. It was explained previously that increasing the drug concentration in the hydration medium could impart more driving force for the drug to be encapsulated into the vesicles resulting in higher EE% [39,40]. Unlike other factors, increasing TMC concentration resulted in lower EE%, which may be related to increased viscosity of the hydration medium resulting in hindering encapsulation of more drugs [41].

All the investigated factors were found to provide a significant effect on the particle size of the formulated EH emulsomes as indicated in Table 3. Higher PC content resulted in a significant decrease in the PS, which is contrary to previous results [42] but in agreement with other results that reported that with more phospholipid content, more emulsomes with smaller diameter are formed [23].

Increasing EH concentration to the lipid ratio led to a significant increase in the EE% and consequently increases the particle size due to increasing the drug content entrapped in the prepared emulsomes.

TMC is a positivly charged polysaccharide. This positive charge causes electrical repulsion among polymeric chains resulting in steric hinderance, which contributes to the size expansion of the particles [43,44], especially when TMC is used as a second layer [45].

The zeta potential value was represented in Table 3. Zeta potential is an important label for the identification of the prepared nanoparticle physical stability. The higher Zp value > 30 mV indicates the higher stability due to increasing the repulsion force between the particles, which can overcome the Van der Waals attractive forces, hence preventing particles aggregation [46]. All prepared formulae have a positive surface charge due to TMC coating and increased significantly by increasing TMC concentration, as in Table 2, with no interaction with either PC: CA molar ratio or EH: T. lipids molar ratio. TMC is positively charged polyelectrolyte at pH 7.4 due to the quaternary ammonium groups. Successful coating of the prepared emulsomes with TMC is due to electrostatic interactions occurring between phospholipids with negative charges and primary amino groups of chitosan with positive charges [47] in addition to other suggested mechanisms such as hydrogen bonding between the polysaccharide and the phospholipid head groups [48].

Higher ZP values observed after increasing the amount of TMC confirm the incorporation of the polyelectrolyte (TMC) in the vesicles’ structure, more possibly as a layer on the surface of the vesicles [49].

Increasing the TMC ratio from 0.25 to 0.5 *w*/*v* resulted in a significant increase in the RT at constant PC: CA and EH: T. lipids ratios. TMC is a mucoadhesive material, its higher water uptake and swelling results in increasing the adhesiveness of the prepared emulsomes and elongated the RT [50]. Unlike the TMC ratio, neither PC: CA nor EH: T. lipids ratio was found to have a significant effect on the retention time of the prepared emulsomes.

Although a higher TMC ratio resulted in longer RT, it resulted in significantly lower Kp at constant PC: CA and EH: T. lipids ratios. This may be related to the fact that interaction between TMC and the phospholipid layer resulted in more stable vesicles, which delayed the drug release and thus decreased the permeability coefficient [51] in addition to the larger vesicle size of emulsomes prepared with higher TMC ratio. Both PC: CA and EH: T. lipids ratios have a positive effect on increasing the permeability coefficient of the prepared emulsomes.

There was an inverse relationship between the vesicular size and permeability coefficient as vesicles with smaller sizes had higher Kp. It has previously been noted that when vesicular size reduces, the attributed surface area: volume ratio increases; indicating that more drugs could be closer to the particle surface, potentially leading to improved drug release and permeation [52].

EH- emulsomes chitosan-coated particles have the ability to regulate EH release, minimize its toxicity, and improve its therapeutic effectiveness. There was an interaction between increasing the PC: CA molar ratio and EH: T. lipids molar ratio, Figure 2. Although the presence of CA as a cationic material could improve the emulsomes stability as a result of charge-induced repulsion between the bilayer surfaces, further increase in the PC: CA molar ratio could disturb the emulsomes lattice structure and produce irregular structure, so as to increase the space for carrying drugs and improve the drug delivery carrier capacity and hence increase the Kp and RT value [53].

#### 3.3.2. Optimization of EH Mucoadhesive Emulsomes

**Figure 3:**
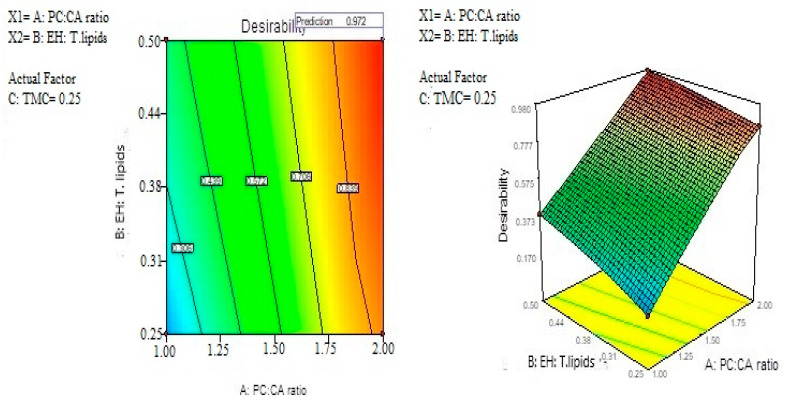
Optimization of EH mucoadhesive emulsomes.

The optimum levels of the variables were predicted by applying response constraints. The computed desirability was 0.972. The prepared optimized formulation was subjected to characterization. No major residual error was found indicating the validity of numerical optimization for this study. The optimized formulation levels are demonstrated in Figure 3. The results indicated that the optimized formula shows nanoparticle size 177.013 nm, EE 79.44%, ZP > 30 mV (32.12 ± 3.28), Kp value = 5.68 cm/h, and RT up to 120 ± 13 s. These results indicated that the optimized EH emulsomes formula No. 10 is the best selected formula.

### 3.4. Morphological Evaluation

**Figure 4:**
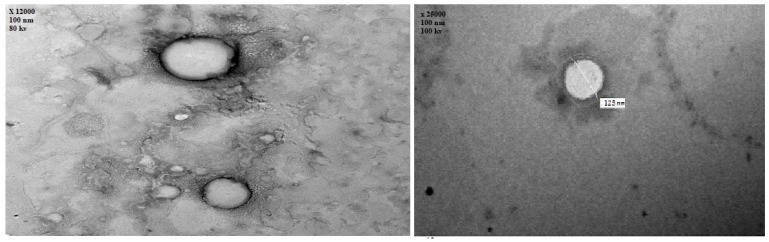
Transmission electron microscope photography of EH emulsomes (×25,000).

Figure 4 illustrates the transmission electron microscope examination of the optimized EH-loaded emulsomes (×25,000). The TEM photographs revealed that most emulsomes particles were nanosized spherical in shape, consisting of a dark phospholipid multilayer around a brighter solid lipid core.

### 3.5. Stability Study of the Optimized Formulation

The capability of emulsomes to maintain the EH entrapment efficiency and to preserve their particle size, during refrigerated storage and at room temperature for three months was assessed. Non-significant difference was found concerning the EE%, particle size, or Zeta potential of the stored formulation (F10) at both conditions, 4 °C and ambient temperature, as shown in Table 5, indicating stability of this formulation and its ability to maintain the drug encapsulated inside the vesicles without observed particle size increase or particles aggregation.

### 3.6. In Vivo Biodistribution Study

**Figure 5:**
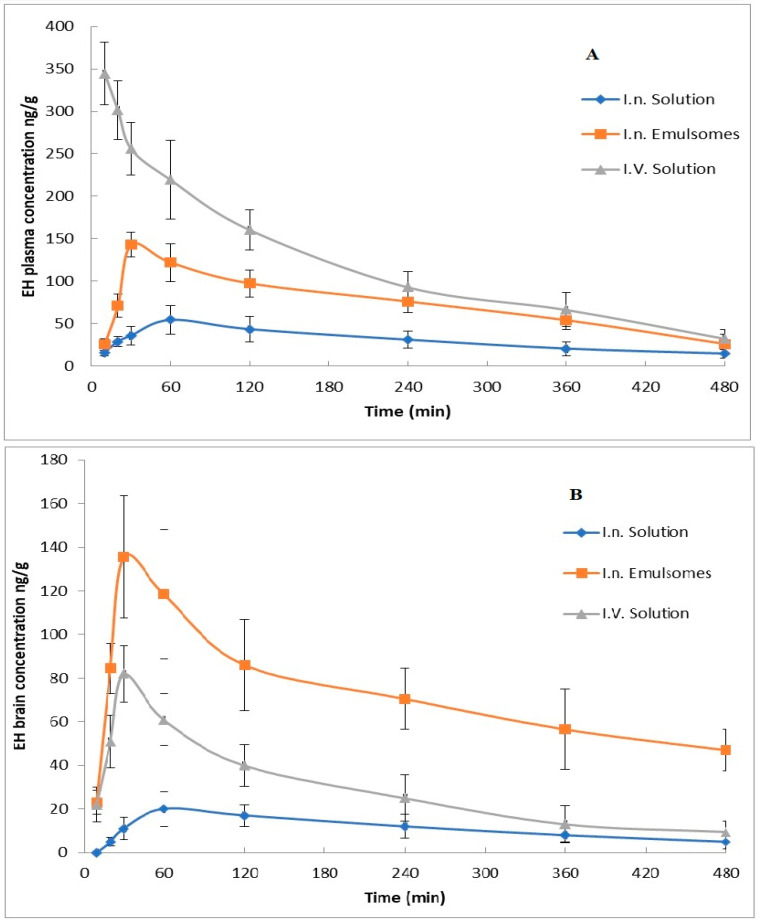
ET concentrations in mice after administration of various formulations: (**A**) plasma concentrations, (**B**) brain concentrations.

EH concentration in mice plasma and brain after administration of different treatments against time is shown in Figure 5, while its pharmacokinetic parameters, as well as DTE% and DTP%, are presented in Table 6. It was noticed that nasal administration of mucoadhesive EH-emulsomes formula has significantly higher C_max_ and AUC_(0–8)_ than i.v and i.n EH solution. The higher drug concentration in the brain after EH-emulsomes administration could be attributed to the nano vesicle size, which allows drug particles to be transported deeper into the olfactory epithelial cells layers [54] and translocated easily from one cerebral compartment to another [55]. The permeation enhancing effect of chitosan and the lipid structure of emulsomes increase the EH permeation affinity through the nasal membrane via the olfactory neurons in the olfactory bulb [55,56].

The shorter T_max_ in the brain than in plasma after EH-emulsomes administration indicated the rapid passage and targeting of the drug to the brain. The previous result could also be proved by the high DTE% and DTP% values. The latter demonstrates the capacity of the EH-emulsomes formula to deliver EH directly to the brain with greater concentrations and a faster onset of action.

Enhanced in vivo bioavailability of EH from the prepared nasal emulsomes can be correlated to the in vitro enhancement of drug permeation through the nasal mucosa. This enhancement is related to high mucoadhesive effect of TMC, which leads to longer residence time with lower mucociliary clearance enabling the emulsomes particles to remain attached to the nasal mucosa, resulting in improved drug permeation [57]. In addition, it was reported that only positively charged chitosan can trigger the opening of tight junctions and thereby facilitate the paracellular transport [58].

The higher DTI and DTP indicate that there is more EH concentrated in the brain rather than in blood plasma, which means that the drug concentration does not depend on drug bio-distribution, but the drug transport is by a direct axonal CNS drug transport [59] and more absorbable and accumulation drug concentration would be delivered directly to the brain. Additionally, a transient effect of chitosan facilitates a higher paracellular contribution and admission through the BBB [60]. The high DTI and DTP values were also related to the EH-emulsomes’ KP value, which means increasing the apparent EH brain permeability and targeting via olfactory and trigeminal regions [61].

### 3.7. Histopathological Study

**Figure 6:**
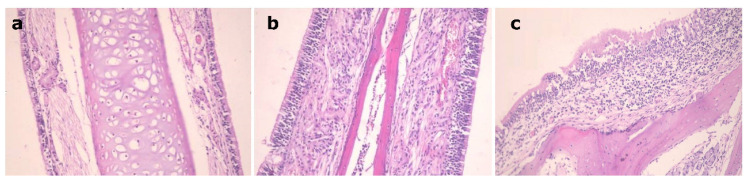
Nasal mucosal tissue of: (**a**): control mouse, (**b**): mouse received EH solution, (**c**): mouse received EH mucoadhesive emulsomes.

Figure 6 shows the nasal mucosal tissue of the control group, Figure 6a, the group that received a nasal solution of the drug, Figure 6b, and the group that received the EH mucoadhesive emulsomes, Figure 6c. Group (c) showed normal tissue features with preservation of the ciliated respiratory epithelium without obvious hyperplasia or necrosis of nasal mucosa in a similar way just as that of the other two groups. This indicates that the EH mucoadhesive emulsomal formulation with its components has no ciliotoxic effect on the nasal mucosa and can be applied safely.

## 4. Conclusions

EH emulsomes were prepared using a thin-film hydration method and the formulations were optimized using full factorial 2^3^ design to evaluate the effects of formulations variable on dependent variables. Prepared EH emulsomes containing phosphatidylcholine: Compritol (CA) molar ratio equal to 2, EH to total lipids molar ratio 0.5, and trimethyl chitosan concentration 0.25% *w*/*v* were spherical in shape with particle size 178.42 ± 21.36 nm, 79.73 ± 2.96 entrapment efficiency percent, and positive zeta value of 32.12 ± 3.28, which facilitate the nose-to-brain targeting. The shorter brain’ Tmax after EH-emulsomes administration indicates rapid targeting of drug to the brain. The higher DTI and DTP facilitates higher paracellular contribution and admission through the BBB, which means increasing the apparent EH brain permeability and targeting with non-ciliotoxic safe effect on the nasal mucosa. The efficient EH concentration in the brain could improve its effectiveness in migraine treatment.

## Figures and Tables

**Table 1:** Full experimental (2^3^) factorial design parameters and constraints.

Independent Variables	Level of Variables Low(−1)–High (1)
X1: PC: CA molar ratio	1–2
X2: EH: T. lipids molar ratio	0.25–0.5
X3: TMC Conc.	0.25% *w*/*v*–0.5% *w*/*v*
Responses	Constraints
Y1: Entrapment Efficiency (EE%)	Maximize
Y2: Particle size (PS)	Minimize
Y3: Zeta potential (ZP)	In-range
Y4: Permeability Coefficient (Kp)	Maximize
Y5: Residence time (RT)	In-range

PC: phosphatidylcholine, CA: Compritol, TMC Conc: trimethyl chitosan concentration.

**Table 2:** Experimental runs, independent and dependent variables of the 2^3^ full factorial experimental designs of EH-loaded emulsomes.

Runs	Factors (Independent Variables)			Responses (Dependent Variables)					
	PC: CA Molar Ratio	EH: T. Lipids Molar Ratio	TMC Conc *w*/*v*	Y1:EE (%)	Y2: PS (nm)	Y3:ZP (mV)	Y4: Kp (cm/h)	Y5: RT (s)	DLC (%)
F1	1:1	0.25: 1	0.25	41.63 ± 3.62	293.86 ± 18.32	26.23 ± 3.26	3.24	123 ± 12	5.4 ± 0.6
F2	1:1	0.25: 1	0.50	35.42 ± 3.84	361.46 ± 22.48	28.61 ± 4.52	2.92	196 ± 16	3.6 ± 0.3
F3	1:1	0.50: 1	0.25	55.78 ± 2.58	325.23 ± 30.57	25.48 ± 4.68	3.91	114 ± 11	13.2 ± 1.2
F4	1:1	0.50: 1	0.50	46.66 ± 3.12	403.24 ± 26.27	27.97 ± 3.89	3.63	208 ± 20	8.9 ± 0.9
F5	1.5:1	0.38: 1	0.38	73.52 ± 2.51	187.12 ± 16.81	32.48 ± 4.87	5.35	195 ± 15	11.6 ± 1.5
F6	1.5:1	0.38: 1	0.38	74.63 ± 1.96	184.52 ± 21.24	32.43 ± 2.95	4.90	203 ± 23	11.3 ± 1.6
F7	1.5:1	0.38: 1	0.38	78.61 ± 2.37	192.24 ± 20.85	33.15 ± 3.50	5.17	176 ± 18	11.7 ± 0.9
F8	2:1	0.25: 1	0.25	65.52 ± 2.82	136.45 ± 14.69	32.14 ± 3.72	5.42	132 ± 16	8.1 ± 0.8
F9	2:1	0.25: 1	0.50	57.48 ± 3.27	167.81 ± 19.27	34.56 ± 4.22	4.61	231 ± 24	5.7 ± 0.4
F10	2:1	0.50: 1	0.25	79.73 ± 2.96	178.42 ± 21.36	32.12 ± 3.28	5.68	120 ± 13	17.8 ± 2.1
F11	2:1	0.50: 1	0.50	66.49 ± 3.08	208.76 ± 18.96	33.92 ± 3.66	4.16	228 ± 19	12.3 ± 1.1

EE: Entrapment Efficiency, PS: particle size, ZP: Zeta potential, Kp: permeability coefficient, RT: Residence time.

**Table 3:** The design expert results of all response variables.

Source	PS (nm)		EE%		ZP		Kp		RT	
	F	*p*-Value	F	*p*-Value	F	*p*-Value	F	*p*-Value	F	*p*-Value
Model	733.64	<0.0001	49.48	0.0043	109.95	0.0013	19.51	0.0168	22.93	0.0133
A: PC: CA molar ratio	3809.33	<0.0001	201.52	0.0008	574.99	0.0002	82.56	0.0028	4.56	0.1223
B: EH: T. lipids molar ratio	193.57	0.0008	59.14	0.0046	4.04	0.1379	3.07	0.1780	0.13	0.7385
C: TMC Conc (mg)	341.54	0.0003	33.55	0.0102	79.47	0.0030	*18.62*	0.0229	130.27	0.0014
AB_3_	0.4479		0.7540		0.5257		0.1038		0.6210	
AC	0.0049		0.4160		0.5691		0.0841		0.3094	
BC	0.4640		0.2896		0.6513		0.3965		0.4274	
Lack of Fit	0.4117		0.7908		0.5867		0.3623		0.7886	
Adequate precision	78.035		22.950		28.941		12.565		11.538	
R^2^	0.9993		0.9900		0.9955		0.9750		0.9787	
Adjusted R^2^	0.9980		0.9700		0.9864		0.9250		0.9360	
Predicted R^2^	0.9861		0.9681		0.9503		0.9662		0.8989	
SD	3.97		2.23		0.36		0.24		11.59	
%CV	1.65		3.64		1.17		5.39		6.62	

**Table 4:** Final equation in terms of the tested factors.

	PS (nm)	EE%	ZP	Kp	RT
Intercept	+259.40	+56.09	+30.13	+4.20	+169.00
A: PC: CA molar ratio	−86.54	+11.22	+3.06	+0.77	+8.75
B: EH: T. lipids molar ratio	+19.51	+6.08	−0.26	+0.15	−1.50
C: TMC Conc (mg)	+25.91	−4.58	+1.14	−0.37	+46.75
A × B	+1.22	−0.27	+0.091	−0.20	−2.25
A × C	−10.49	−0.74	−0.081	−0.22	+5.00
B × C	+1.17	−1.01	−0.064	−0.084	+3.75

EE: Entrapment Efficiency, PS: particle size, ZP: Zeta potential, Kp: permeability coefficient, RT: Residence time.

**Table 5:** Stability study of the optimized EH-loaded emulsomes formulation (F10).

	EE%	PS (nm)	ZP (mV)
At zero time	79.73 ± 2.96	178.42 ± 21.36	32.12 ± 3.28
After 3 months stored at 25 ± 2 °C and controlled humidity of 75%	78.85 ± 2.06	180.92 ± 19.19	32.44 ± 1.88
After 3 months stored at 25 ± 2 °C and controlled humidity of 75%	78.63 ± 1.96	183.12 ± 1.36	31.92 ± 2.14

**Table 6:** EH hydrobromide pharmacokinetic parameters in plasma and brain.

Formula	C_max_ (ng/g)		T_max_ (min)		AUC_(0–8)_ (ng/g.hr)		DTI	DTE%	DTP%
	Plasma	Brain	Plasma	Brain	Plasma	Brain			
EH emulsomes	143 ± 22 ^#^	271 ± 56 *^#^	60	30 ^#^	588 ± 103 *^#^	582 ± 112 *^#^	4.1 ± 0.35 ^#^	407.6 ± 35.7 ^#^	75.5 ± 4.8 ^#^
i.n EH solution	55 ± 17	85 ± 18 *	60	60	243 ± 56 *	90 ± 23 *	1.5 ± 0.22	152.4 ± 22.4	34.4 ± 1.6
i.v. EH solution	-	189 ± 38	-	30	950 ± 89	231 ± 41	-	-	-

Cmax and AUC _(0_–_8)_ results are recorded as mean SD, *n* = 4, * Significant difference from the i.v. solution at *p* < 0.05, ^#^ Significant difference from the i.n. solution at *p* < 0.05.

## Data Availability

No new data were created or analyzed in this study.
